# 

**Published:** 1871

**Authors:** 


### CONTENTS.

PAGE.

Clinical Lecture on Acute Albuminuria. By Wm. Abram Love, M. D., Atlanta, Ga. . . . . 193
Triplets, with Dropsy of the Amnion. By Edwin S. Ray, M. D., Atlanta, Ga 199
Injury to the Lower Bowel by the Accidental Introduction of the Handle of a Hoe into the rectum. By R. W. Hargadine, M. D., Felton, Del. 202
Notes on New Remedies. By John Stainback Wilson, M. D., Atlanta, Ga. 204
Cases from the Surgical Clinic of Atlanta Medical College. By W. F. Westmorelane, Atlanta, Ga . . . . . 209
Medical Clinic of Atlanta Medical College. Service of Dr. J. G. Westmoreland, Atlanta, Ga. . . . . 213
Extracts from the Minutes of Atlanta Academy of Medicine. . . . . 216
Atlanta Academy of Medicine. Edward P. Ingraham, M. D., Atlanta, Ga 221
Electrolysis, and its application to the treatment of Disease. By A. D. Rockwell, M. D, New York. . . . . 229
Cases of Cancer Treated with Cundurango. By D. W. Bliss, M. D., Washington, D. C. . . . . 240
On a Rare Form of Poisoning by Quinine. By A. Brayton Ball, M. D. 244
Combined Enucleation and Avulsion of a Fibrous Tumor of the Uterus. By J. Matthews Duncan, M. D. . . . . 248
Case of Excision of the Lateral Half of the Tongue. By George Buchanan, A. M, M. D. . . . . 250
Kennedy's Concentrated Extract of Pinus Canadensis. By J. Marion Sims, M. D . . . . . 255
Subcutaneous Injections of Arsenious Acid in Skin Diseases. . . . . 256
Arsenic in Irritative Dyspepsia. . . . . 256
Remarks on the Pathology and Treatment of Stricture of the Urethra. By W. F. Teevan, B. A., F. R. C. S. . . . . 257
Meigs and Mason (Ohio) Academy of Medicine. . . . . 260
Compound Presentation. . . . . 263
On the Action of Light in Small-pox. By J. H. Waters, M. B. . . . . 265
Temperature During Pregnancy, Labor and Childbed. By S. Brandeis, M. D., Louisville, Ky . . . . . 268
A Case of Perforating Ulcer of the Stomach; Recovery. By T. Tinley, L. R. C. P., &c. . . . . 269
Syphilis of the Nervous System. By E. L. Keys, M. D. . . . . 271
On Lithia. . . . . 272
On Uterine Compression as a Means of Completing Delivery. By Dr. G. Chantreuil, Paris. . . . . 273
On Surgical Dressings. By T. Hiron Bartleet, M. B., M. R. C. S. . . . . 277
On some Disorders of the Nervous System in Childhood. By Charles West, M. D. . . . . 280
A Mirror of the Practice of Medicine and Surgery in the Hospitals of London. . . . . 282
A New Method of Delivering the After-Coming Head in Contracted Pelvis. By William Goodell, M. D. . . . . 283
Control of Hæmorrhage by Ipecac. By William Martin, M. D., M. R. C. S., Billingsville Indiana. . . . . 285
Tetanus—Treated by Hydrate of Chloral—Recovery. By W. M. McGalliard, M. D., Donaldsonville, Louisiana. . . . . 286
The Management of the Placenta. . . . . 288
On the use of the Ecraseur for the Removal of Nævoid Growths. By James F. West, F. R. C. S. . . . . 289
Operations by Students. . . . . 290
A New Departure. . . . . 291
Atlanta Medical College . . . . . 293
Cundurango. . . . . 294
Yellow-Fever . . . . . 295
The late Dr. D. C. O'Keefe. . . . . 296
Our Journal . . . . . 298
Dislocation and Fracture of Femur. . . . . 298
Dactylitis Syphilitica. . . . . 299
Orthopædic Apparatus . . . . . 300
Miscellaneous. . . . . 300

